# A hybrid intensive care unit in a resource-constrained cancer hospital in northeast India

**DOI:** 10.5588/pha.26.0009

**Published:** 2026-05-18

**Authors:** A. Kesha, R.S. Tayshetye, P. Mukherjee, H.D. Shewade, M. Das, M.B. Lokesh, S.G.S. Kumar, D. Raman, R. Kannan

**Affiliations:** 1 Cachar Cancer Hospital and Research Centre (CCHRC), Silchar, India;; 2 ICMR National Institute of Epidemiology (ICMR-NIE), Chennai, India;; 3 FIND, Delhi, India;; 4 Cloudphysician Healthcare Pvt. Ltd., Bengaluru, India.

**Keywords:** ICU, digital health, malignancy, SORT IT, telehealth, remote monitoring

## Abstract

**BACKGROUND:**

To compensate for the non-availability of intensive care specialists, Cachar Cancer Hospital and Research Centre (a tertiary hospital in a resource-constrained setting, northeast India) introduced a hybrid intensive care unit (ICU with a centralised monitoring model).

**METHODS:**

This was a cohort study involving secondary data for patients admitted with curative intent in the hybrid ICU (October 2021 to December 2022). We report on our early implementation experience in the form of patient management and outcome indicators.

**RESULTS:**

There were 909 admissions with curative intent, of which 73% were post-surgical. There were 238 admissions from October 2021 to March 2022, which increased to 671 from April to December 2022 (the period when effective implementation happened). The changes (unadjusted *P* value) in management and outcome indicators over these periods were ventilator use, 8%–12% (*P* = 0.128); death during admission, 7%–6% (*P* = 0.575); readmission within 3 months, 21%–25% (*P* = 0.309); and median length of stay, 1–2 days (*P* = 0.035).

**CONCLUSION:**

Despite an increase in admissions following the introduction of the hybrid ICU, it did not adversely impact management and outcome indicators. Post-ICU hospital care, patient caregiver education, and home care team engagement should be strengthened to decrease readmissions.

Cancer care has improved in recent decades with an increase in overall survival. Patients may be admitted to the intensive care unit (ICU) for both cancer-related and noncancer-related morbidities.^1^ In a European multicentre study (2008), 15% admissions were cancer-related.^2^ ICU readmissions were associated with increase in mortality and poor performance status when compared to non-readmission patients.^3^ Continuous monitoring is needed while in ICU care. People with cancer who require ICU support have high mortality if the cancer- and noncancer-related morbidities are not managed in a timely manner.^4^ In resource-constrained settings, due to lack of trained manpower (intensivist), adequate and continuous monitoring may not be possible. Shortage of intensivists can lead to increase in mortality.^5,6^ The hybrid ICU or tele-ICU concept started in 1970 with video connection made between the patients’ beds and a remotely available intensivist.^5^ With advances in technology and the internet, high precision cameras are available for sharing information in real-time. Tele-ICU implementation was associated with a reduction in mortality, especially among medium- and high-risk patients, and less on-site physician duties pertaining to electronic medical records.^7,8^ ICU length of stay for critically sick adult patients was not decreased by telemedicine-assisted daily multidisciplinary rounds led by a board-certified intensivist.^9^

There is limited published literature on the use of hybrid ICU in India, especially in the context of cancer in a resource-constrained setting. Cachar Cancer Hospital and Research Centre (CCHRC) is a comprehensive cancer care facility located in the outskirts of Silchar town (Cachar district) in the Barak Valley of southern Assam, Northeast India (https://doi.org/10.6084/m9.figshare.32051229). Due to lack of intensivists, hybrid ICU is being used at CCHRC since October 2021. The hybrid ICU works on 24-hour virtual monitoring of each patient. The concerned doctor evaluates the patient; the nursing staff updates all information on a virtual system where a team of critical care intensivists over cloud (Cloudphysician® at CCHRC) has access.^4–10^ At CCHRC, the Cloudphysician® reviews the patient and the data and advises action to be taken. There has been no formal assessment of whether the continuous monitoring and timely interventions under hybrid ICU in resource-constrained cancer hospital settings of India have contributed towards better management and outcomes over time in terms of ventilator use, ICU readmissions, and deaths (as admission outcomes). With the introduction of hybrid ICUs, ventilator use is expected to increase with reduction in deaths during admission and reduction in readmissions in near future.

We therefore aimed to document the changes in patient management and outcome during the implementation of a hybrid ICU in our setting. Among patients admitted with curative intent in the hybrid ICU of CCHRC Silchar (October 2021 to December 2022), we described the trends and compared over time, the i) proportion receiving ventilatory support; ii) proportion of deaths during admission; iii) occurrence of readmission within 3 months; and iv) median length of stay.

## METHODS

This was a cohort study involving secondary data. CCHRC started in 1996 and is administered by the Cachar Cancer Hospital Society, a not-for-profit non-government organisation, and caters to people with cancer in the northeast region of India. Every year, the hospital sees more than 25,000 follow-up visits and more than 4,000 new patients. Nearly 80% of patients are daily wage workers and agricultural and tea garden labourers, and 75% are treated for free or at subsidised charges. In addition to hospital-based clinical care, services also include home-based care, telemedicine centres, satellite clinics, community-based screening, and awareness programmes.

### Hybrid ICU

Hybrid ICU is the platform for coordinated patient management. CCHRC team manages overall care, including surgery, long term management, internal medicine, and medical oncology. The virtual team (Cloudphysician®) provides augmented monitoring and critical care input and maintains the patient medical data in the Cloudphysician® database. The 15 bedded ICU at CCHRC is based on a centralised monitoring model involving a technical hub in Bengaluru. It involves real-time collection and delivery of continuous (24/7) clinical data streams, including vital signs, laboratory, scans, and ventilator management. The Cloudphysician® team augments bedside monitoring with high-definition cameras (n = 4–5). To enhance treatment, the bedside nursing team works with the virtual nursing team. Data are processed by intensivist-led team (n = 3–4, including doctors and nurses) at Cloudphysician® who provide inputs on critical care, while surgical oncology, internal medicine, family communication, and procedures are provided by the bedside.

**FIGURE 1.**
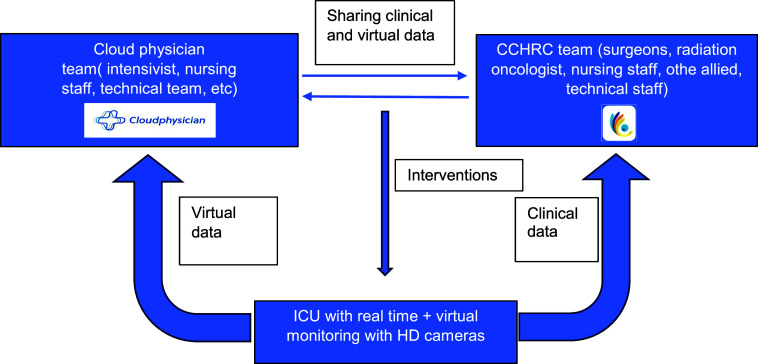
Hybrid ICU working flow chart at CCHRC Silchar, Northeast India. CCHRC = Cachar Cancer Hospital and Research Centre; ICU = intensive care unit.

Prior to the hybrid ICU, the surgical intensive care was looked after by surgeons in turns with the ICU nurses. Often the burden of decision making would fall on the single doctor on call. With the introduction of hybrid ICU, twice a day, joint clinical case discussions are conducted. Medical team based at hybrid ICU and Cloudphysician® discuss the admitted patient status and the necessary interventions. Apart from this, if any immediate intervention is required, the Cloudphysician® intimates (through speakers) the nursing and medical staff based at the hybrid ICU (Figure 1). CCHRC nursing team had difficulties in using technology in the initial implementation period; eventually, the nursing staff learnt how the technology works.

For patients discharged from the ICU and the hospital, there is a home care team within CCHRC that visits the patient’s home on a case-to-case basis. They provide home-based post-operative care and pain management care, and educate patient and family regarding tube feed, stoma care, and other related care.

### Study population and data

We included all patients with cancer admitted in hybrid ICU (with curative intent) at CCHRC Silchar between October 2021 and December 2022. Patients admitted before October 2021 in the ICU and readmitted during the study period were excluded. The following individual level variables were extracted from electronic Cloudphysician® database: unique hospital ID, date of admission, age, gender, type of cancer, post-surgical admission (yes/no), patient severity score at admission (APACHE II – acute physiology and chronic health evaluation), ventilator support received during admission, including non-invasive and invasive (yes/no), type of ventilator support (intermittent mandatory ventilation or non-invasive invasive), whether patient got readmitted within 3 months (yes/no), admission outcome, and date of admission outcome.

### Statistical analysis

Microsoft Excel was used to extract the data, and EpiData 2.2.2.183 (EpiData Association, Odense, Denmark) and STATA (version 12.1, copyright 1985-2011 StataCorp LP USA, serial number: 30120504773) were used for analysis. We have described the total number of patients admitted and among them the number (proportion) receiving ventilatory support and with death as the admission outcome. We calculated the number (proportion) getting readmitted in the ICU within 3 months. Among those who were discharged successfully, we calculated the median (interquartile range, IQR) admission duration. We presented the trend of these indicators by quarterly-admission cohorts from October 2021 to December 2022. We have described the total number of patients admitted and among them the numbers (proportion) receiving ventilatory support and with death as the admission outcome. We calculated the number (proportion) getting readmitted in the ICU within 3 months. Among those who were discharged successfully, we calculated the median (IQR) length of stay. We presented the trend of these indicators by quarterly-admission cohorts from October 2021 to December 2022. When compared to the October 2021 to March 2022 admission cohort, we assessed the change in the above indicators in April to December 2022 admission cohort (overall and stratified by post-surgical admission). Over the first two quarters (October 2021 to March 2022), the nurses would always confirm the recommendations from the Cloudphysician® with the inhouse physician. Hence, October 2021 to March 2022 admission cohort was considered as a baseline for comparison with April to December 2022. We did not have the data routinely documented before October 2021 to enable a before-during comparison. For unadjusted comparisons between proportions, χ² test was used. For unadjusted comparison between medians, Mann–Whitney *U* test was used. The overall comparisons were also adjusted for differences in age, gender, type of cancer, post-surgical admission (yes/no), and APACHE II score using modified Poisson regression with robust variance estimates.

### Ethical statement

Ethics approval was obtained from Institution Review Board, CCHRC Silchar, India (ECR/925/Inst/AS/2017/RR-21, dated 13 April 2024). As the study involved review of anonymous patient records (electronic secondary data), a waiver for written informed consent was approved by the ethics committee.

## RESULTS

A total of 909 patients were admitted in the hybrid ICU with curative intent. There was an increase in absolute quarterly admissions: average of 119 per quarter during October 2021 to March 2022 and 224 per quarter during April to December 2022. Of the 909 patients, the mean age was 50.6 years (standard deviation 16.4) and 476 (52%) were males. The common types of cancers were head and neck (n = 293, 32%) and gastrointestinal (n = 246, 27%). Post-operative patients made up 659 (73%) of the admissions, and the median APACHE II score was 9 (IQR 6, 13) – see Table 1.

**TABLE 1.** Characteristics of patients admitted in hybrid ICU with curative intent in a tertiary hospital, Silchar, Northeast India, October 2021 to December 2022.

Characteristics	N (%)
Total	909 (100.0)
Demographic characteristics	
Age in years	
0–14	41 (4.5)
15–24	24 (2.6)
25–34	58 (6.4)
35–44	129 (14.2)
45–54	220 (24.2)
55–64	242 (26.6)
≥65	195 (21.5)
Patient’s gender	
Men	476 (52.4)
Women	433 (47.6)
Clinical characteristics	
Type of cancer	
Head and neck	293 (32.2)
Gastrointestinal	246 (27.1)
Breast and gynae	164 (18.0)
Haematological	39 (4.3)
Male reproductive system	42 (4.6)
Skin and cutaneous	31 (3.4)
Urinary system	39 (4.3)
Others^A^	47 (5.2)
Missing	8 (0.9)
Post-surgical admission	
Yes	659 (72.5)
No	173 (19)
Missing	77 (8.5)
APACHE II score	
0–9	505 (55.6)
10–19	310 (34.1)
20–29	72 (7.9)
≥30	19 (2.1)
Missing	3 (0.3)
Mean (SD)	10.3 (6.9)
Treatment-related characteristics	
Readmission within 48 h	12 (1.3)
Ventilator support received during admission	101 (11.1)
Type of ventilator support	
Intermittent mandatory ventilation	79 (8.7)
Non-invasive	22 (2.4)
Not applicable	808 (88.9)
Readmission within 3 months	217 (23.9)
Admission outcome	
Successful discharge from ICU	769 (84.6)
Death	58 (6.4)
Leave against medical advice	74 (8.1)
Transfer to other/higher centre	8 (0.9)

ICU = intensive care unit; APACHE = acute physiology and chronic health evaluation.

A Others include central nervous system (n = 8), musculoskeletal (n = 27), and thorax (n = 12).

**FIGURE 2.**
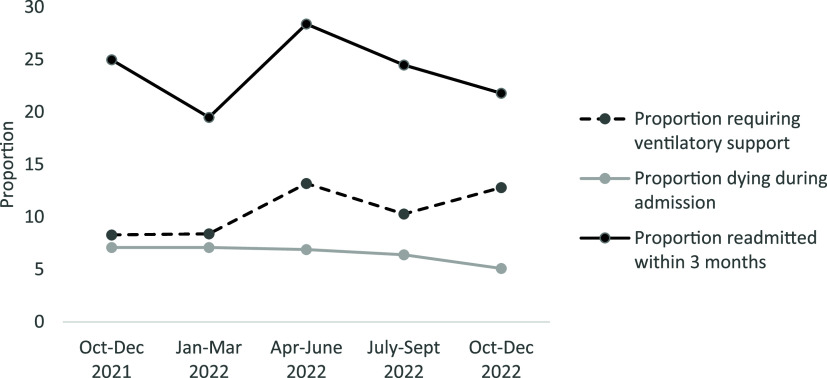
Quarterly trends of key management and outcome indicators at the hybrid ICU of CCHRC Silchar, Northeast India, October 2021 to December 2022. CCHRC = Cachar Cancer Hospital and Research Centre; ICU = intensive care unit.

Ventilator was used in 101 (11%) admissions (of these, 40 died). A total of 58 (6%) patients died during admission and 217 (24%) were readmitted within 3 months. The median length of stay for individuals who were successfully discharged was 2 days (IQR 1, 4). The trends of these indicators across quarters are shown in Figure 2. All these indicators were lower among post-surgical admissions when compared to other admission (see Table 2).

**TABLE 2.** Changes in key management and outcome indicators among patients admitted in hybrid ICU with curative intent in a tertiary hospital, Silchar, Northeast India, October 2021 to December 2022 (N = 909).

Outcomes^A^	Post-surgical admissions	Yes	Other admissions	Yes	All	Yes
		n (%, 95% CI)		n (%, 95% CI)	N	n (%, 95% CI)
Provided ventilatory support^B^	659	52 (7.8)	173	43 (24.9)	909	101 (11.1)
October 2021 to March 2022	156	10 (6.4, 3.1–11.5)	35	6 (17.1, 6.6–33.7)	238	20 (8.4, 5.2–12.7)
April to December 2022	503	42 (8.3, 6.1–11.1)	138	37 (26.8, 19.6–35.0)	671	81 (12.0, 9.7–14.8)
Deaths during admission^B^	659	14 (2.1)	173	35 (20.2)	909	58 (6.4)
October 2021 to March 2022	156	5 (3.2, 1.1–7.3)	35	7 (20.0, 8.4–36.9)	238	17 (7.1, 4.2–11.2)
April to December 2022	503	9 (1.8, 0.8–3.4)	138	28 (20.3, 13.9–28.0)	671	41 (6.1, 4.4–8.2)
Readmitted within 3 months^B^	659	134 (20.3)	173	61 (38.7)	909	217 (23.9)
October 2021 to March 2022	156	33 (21.2, 15.0–28.4)	35	9 (25.7, 12.5–43.3)	238	51 (21.4, 16.4–27.2)
April to December 2022	503	101 (20.1, 16.9–24.1)	138	52 (37.7, 29.6–46.3)	671	166 (24.7, 21.5–28.2)
		Median (IQR)		Median (IQR)		Median (IQR)
Admission duration^C^	587	2 (1, 4)	122	3 (2, 6)	769	2 (1, 4)
October 2021 to March 2022	138	1 (1, 4)	24	3 (2, 6)	200	1 (1, 4)
April to December 2022	449	2 (1, 4)	98	3 (2, 6)	569	2 (1, 4)

ICU = intensive care unit; CI = confidence interval; IQR = interquartile range.

A No statistically significant difference in the outcome between the two time periods (overall and stratified by post-surgical admission) after adjusting for age, gender, cancer type, type of admission, and APACHE II score.

B 95% CIs are for comparison between the two time periods.

C Among those successfully discharged.

When compared to initial two quarters of implementation, in April to December 2022, the proportion receiving ventilatory support increased (8%–12%), and the proportion of deaths during admission reduced (7%–6%); however, this was not statistically significant. The proportion of readmissions within 3 months increased from 21% to 25%, though not statistically significant (see Table 2). The median length of stay doubled from one to two days; this was statistically significant on unadjusted analysis, but the association disappeared after adjusting for age, gender, cancer type, type of admission, and APACHE II score (see Table 2).

## DISCUSSION

During the use of hybrid ICU in CCHRC, Silchar (October 2021 to December 2022), although the absolute number of admissions increased, there was no significant change in the proportion receiving ventilatory support, death rate during admission, readmission within 3 months, and median length of stay. Though there were no significant changes in patient management and outcome, it was encouraging that the indicators did not worsen. Use of hybrid ICU in a resource-constrained tertiary cancer hospital relieves the surgical oncologists to perform their routine oncology role. Previously, before the use of hybrid ICU, they used to manage the ICU in person. Overall, the median length of stay in post-surgical admissions increased by 1 day (absolute baseline 1 day), for other admissions there was no change (median 3 days). The length of stay appears to be adequate for post-surgical and other admissions, respectively. Hence, other reasons for high readmissions like suboptimal patient care once patients are discharged from ICU and post-operative complications should be explored.

There were limited studies on hybrid ICU managing cancer patients with which to compare our findings and no studies from India. The death rate among other admissions (other than post-surgical) in our hybrid ICU was 20%, which is lower than reported globally in normal ICUs (not hybrid) through a systematic review published in 2022.^11^

The following are the implications of our study. Hybrid ICU may be continued as the trend of change in the management and outcome indicators is towards the desired direction (though not significant). The hospital care (post ICU), patient caregiver education, and home care team engagement should be strengthened to decrease high readmissions. This study depicts the initial 1-year use of hybrid ICU. For comparisons in the future, these data can be retained as a baseline. Other future research may include exploring the perspectives of nurses delivering care under the guidance of Cloudphysician®.

The strengths in this study are that this is the first operational research to assess changes in management and outcome indicators of a hybrid ICU in a tertiary cancer centre in a resource-constrained setting of northeast India. Future studies, especially from CCHRC, may use this as a baseline. Limitations included absence of a comparison arm and a before–during comparison. Therefore, this is neither an effectiveness study nor an impact evaluation.

## CONCLUSION

Although admissions increased over time, we did not find any significant changes in the management and outcome indicators. Introduction of hybrid ICU did not adversely impact management and outcome indicators. The trend of these indicators is in the desired direction (except for readmissions). To decrease readmission rates, post-ICU and hospital discharge care could be improved by educating patients/their caregivers and strengthening home-based follow-up and care.

## References

[1] AbuSara AK, Nazer LH, Hawari FI. ICU readmission of patients with cancer: incidence, risk factors and mortality. J Crit Care. 2019;51:84-87. 30771692 10.1016/j.jcrc.2019.02.008

[2] Taccone FS, Characteristics and outcomes of cancer patients in European ICUs. Crit Care. 2009;13(1):R15. 19200368 10.1186/cc7713 PMC2688132

[3] Rodrigues CM, Admission factors associated with intensive care unit readmission in critically ill oncohematological patients: a retrospective cohort study. Rev Bras Ter Intensiva. 2016;28(1):33-39. 27096674 10.5935/0103-507X.20160011 PMC4828089

[4] Ramnath VR, Centralized monitoring and virtual consultant models of tele-ICU care: a systematic review. Telemed J E Health. 2014;20(10):936-961. 25226571 10.1089/tmj.2013.0352

[5] Ries M. Tele-ICU: a new paradigm in critical care. Int Anesthesiol Clin. 2009;47(1):153-170. 19131758 10.1097/AIA.0b013e3181950078

[6] Ramakrishnan N, Breaking barriers to reach farther: a call for urgent action on tele-ICU services. Indian J Crit Care Med. 2020;24(6):393-397. 32863629 10.5005/jp-journals-10071-23447 PMC7435096

[7] Fusaro MV, Becker C, Scurlock C. Evaluating tele-ICU implementation based on observed and predicted ICU mortality: a systematic review and meta-analysis. Crit Care Med. 2019;47(4):501-507. 30688718 10.1097/CCM.0000000000003627

[8] Watanabe T, An evaluation of the impact of the implementation of the Tele-ICU: a retrospective observational study. J Intensive Care. 2023;11(1):9. 36882878 10.1186/s40560-023-00657-4 PMC9989570

[9] Pereira AJ, Effect of tele-ICU on clinical outcomes of critically ill patients: the TELESCOPE randomized clinical trial. JAMA. 2024;332(21):1798-1807. 39382244 10.1001/jama.2024.20651 PMC11581649

[10] Arora VK, Chachra V. Virtual ICU and E-learning tools: scope in critical care medicine in India. Indian J Crit Care Med. 2012;16(3):148-150. 23188955 10.4103/0972-5229.102084 PMC3506072

[11] Nazer LH, A systematic review and meta-analysis evaluating geographical variation in outcomes of cancer patients treated in ICUs. Crit Care Explor. 2022;4(9):e0757. 36119395 10.1097/CCE.0000000000000757 PMC9473777

